# Network Pharmacology and Molecular Docking Analysis on Molecular Targets and Mechanisms of Bushen Hugu Decoction in the Treatment of Malignant Tumor Bone Metastases

**DOI:** 10.1155/2022/2055900

**Published:** 2022-11-16

**Authors:** Tianqing Sang, Tengfei Zhang, Juntao Wang, Yuling Zheng

**Affiliations:** ^1^The First Clinical Medical College, Henan University of Chinese Medicine, Zhengzhou, 450000 Henan Province, China; ^2^Cancer Center, The First Affiliated Hospital of Zhengzhou University, Zhengzhou 450000, China; ^3^Gastroenterology Ward II, The Third Affiliated Hospital of Henan University of Chinese Medicine, Zhengzhou 450000, China

## Abstract

**Purpose:**

To explore the active compounds of the Chinese medicine prescriptions of Bushen Hugu Decoction (BHD) and demonstrate its mechanisms against malignant tumor bone metastasis (BM) through network pharmacology and molecular docking analysis.

**Methods:**

The main components and targets of BHD were retrieved from the TCMSP database, and the targets were normalized by UniProt. The Herbs-Components-Targets network of BHD was established by Cytoscape. The main BM targets were obtained from GeneCards, TTD, DrugBank, and OMIM. STRING and Cytoscape were used to construct a PPI network and obtain hub genes. DAVID and Metascape were used for GO and KEGG enrichment analyses. According to the network topology parameters, the top 4 components were selected for molecular docking verification with the core targets.

**Results:**

Compound–target network of BHD mainly contained 51 compounds and 259 corresponding targets including 107 BHD-BM targets. PPI interaction network and subnetworks identified ten hub genes. GO enrichment analysis found 1970 terms (*p* < 0.05), and 164 signaling pathways (*p* < 0.05) were found in KEGG, including PI3K-Akt signaling pathway, proteoglycans in cancer, prostate cancer, MAPK signaling pathway, and IL-17 signaling pathway. Molecular docking analysis showed that the active components of BHD, quercetin, luteolin, kaempferol, and aureusidin have good binding activity to the core targets.

**Conclusion:**

The potential molecular target and signaling pathways were found for BHD major active components. It provides guidance for the future mechanism research of the BHD in malignant tumor bone metastasis. This study also established the foundation for the new strategy for the pharmacology study of Chinese medicine.

## 1. Introduction

Cancer incidence increased worldwide. Cancer, especially malignant tumor, ranks as the disease with the highest fatality rate after cardiovascular disease globally [1]. Most malignant tumor patients do not die from the primary disease directly but from complications such as cardiovascular disease, infection, secondary primary tumor, and metastases [2]. In recent years, with the development of preventive medicine, early diagnosis, and therapeutic regimens, the survival rate of cancer patients has improved. From 1999 to 2015, the overall cancer death rates decreased by 1.8% per year among men and by 1.4% per year among women [3]. However, the treatment for patients at the advanced stage with multisite metastasis is still very challenging. Metastasis is a common cause of death in malignant tumors. Bone is one of the most common sites of malignant tumor metastatic after the lung and liver. Bone metastases occur in approximately 70% of prostate cancer or breast cancer patients and 30-40% of lung cancer patients [4–6]. Bone metastases can lead to skeletal-related events (SREs), including hypercalcemia, pathological fractures, spinal cord compression, and bone pain. Those devastating SRE symptoms in cancer patients greatly impaired the quality of life, reduced survival, and increased economic burden on patients [7–10].

The standard cares for bone metastases in cancer patients are bisphosphonates and denosumab. Bisphosphonates, the inhibitors of osteoclast activity, can inhibit tumor growth by blocking prenylation. However, clinical studies showed minimal efficiency on tumors due to limited uptake at tumoral sites [11]. Denosumab is a monoclonal antibody against the receptor activator of nuclear factor-kappa-*Β* ligand (RANKL). The RANK/RANKL/OPG axis is critical for the regulation of bone formation and resorption. Inhibition of RANKL may serve as an ideal therapeutic strategy for excessive bone resorption [12]. However, whether long-term use of denosumab has side effects on bone tissues and the immune system is still unknown, further research is needed [13]. Standard care combined with radiation has become a new strategy for the treatment of BM. Unfortunately, this treatment strategy is only partially effective and is palliative, not curative [14, 15].

Chinese medicine is a traditional treatment method original to China. A variety of Chinese medicine prescriptions, herbs, and extracts have been confirmed with the potential to treat malignant tumors by experimental studies and clinical trials [16]. Combining ancient wisdom and modern medical science, Chinese medicine has become an essential part of the comprehensive treatment of malignant tumors in China and several other Asian countries [17]. However, there are few studies on treating bone metastases using Chinese medicine. Bushen Hugu Decoction (BHD) was developed by Professor Zheng Yuling at the Henan University of Chinese Medicine, China. It has achieved good clinical efficacy in the treatment of bone metastases and has obtained a Chinese national patent (No. ZL 201811276433.4). BHD consists of eight natural herbs Cuscutae Semen, Rehmanniae Radix Praeparata, Eucommiae Cortex, Dipsaci Radix, Cistanches Herba, Drynariae Rhizoma, Sinapis Semen, and Radix Angelicae Biseratae. Studies have shown that 7-hydroxycoumarin, the extract of Angelicae Biseratae and Eucommiae, can induce apoptosis of lung cancer cell and prevent kidney damage caused by platinum-based chemotherapy drugs [18, 19].

Cistanches Herba extract phenylethanol glycosides increase the sensitivity of liver cancer patients to oxaliplatin by inhibiting HIF-1*α* signaling pathway [20].

Cuscutae Semen, Dipsaci Radix, Drynariae Rhizoma, and Eucommiae Cortex can inhibit bone destruction through the immune system and inflammation-related pathways [21–24]. In this study, we used network pharmacology technology to investigate the potential mechanism of BHD on bone metastases and molecular docking technology to screen the major effective components and targets for BHD.

## 2. Materials and Methods

### 2.1. Construction of BHD Main Active Component Database and Target Prediction

Traditional Chinese Medicine Systems Pharmacology Database and Analysis Platform (TCMSP, https://old.tcmsp-e.com/index.php) was used to authenticate chemical composition of various herbs in BHD. TCMSP consists of 499 Chinese herbs registered in the Chinese Pharmacopoeia, with 29,384 ingredients, 3,311 targets, and 837 associated diseases [25]. TCMSP is built on the framework of systems pharmacology for herbal medicines to ensure the confidence of results. The toxic pharmacokinetic property parameters were set as follows: oral availability (OB) ≥ 30%, drug-likeness (DL) ≥ 0.18, and half-life (HL) ≥ 4 [26]. The filtered active components were used to screen their protein targets. The targets of active compounds were searched through PubMed, CNKI, and Wanfang databases. Protein target gene names were canonicalized through the UniProt database (https://www.uniprot.org). “Herbs-components-targets” network of BHD was constructed based on network topology parameters (NTP) through Cytoscape 3.7.2. The larger the node, the more targets associated with it, and the more likely it will be a key ingredient in BHD.

### 2.2. Construction of the BHD-BM Gene PPI Interaction Network

The bone metastases-related targets were identified in the GeneCards database (https://www.genecards.org), OMIM database (http://www.omim.org), TTD database (http://bidd.nus.edu.sg/group/cjttd), and DrugBank database (https://www.Drugbank.ca) using “Bone Metastasis”, “Bone Metastases”, “Osseous Metastasis”, “Bone Metastasis of Malignant Tumor” and “Metastatic Carcinoma of Bone” as keywords. The targets were supplemented through literature search. Use Venn script in R language to get the intersection of the targets of BHD components and BM. BHD-BM genes were uploaded to STRING11.0 (https://string-db.org) to build a PPI interaction network. Initial screening of the BHD-BM gene by setting the degree score. The data were corrected by setting the biological species to 'Homo sapiens'. The PPI interaction network of BHD-BM genes was visualized with Cytoscape using 0.9 as the highest confidence level. Topological analysis of BHD-BM genes by Cytohubba in Cytoscape to obtain the hub genes [27].

### 2.3. GO and KEGG Enrichment Analyses

GO and KEGG enrichment analyses of BHD-BM genes were performed using DAVID (https://david.ncifcrf.gov/) online analysis tool and Metascape platform (http://metascape.org/gp/index.html). A *p* < 0.05 was used to screen BHD-BM-related biological process (BP), molecular function (MF), and cellular component (CC). Then, the top ten results were chosen for visualization. Similarly, a *p* < 0.05 was used to screen KEGG pathway analysis. BHD compounds, BHD-BM genes, and top 20 model pathways were used in KEGG enrichment analysis. The NTP (degree, betweenness, and closeness) was analyzed by the built-in tool of Cytoscape to build the BHD compound, BM target, and pathway network.

### 2.4. Active Component-Target Docking

Molecular docking is a well-established computer-based structural method for structurally docking small molecules to target proteins and assessing their binding affinity [28]. It is widely used in new drug discovery. Negative docking energy indicates that the small molecule can bind effectively and autonomously to the target protein [29]. The lower the binding energy, the tighter the bond, and the more effective it is. In this study, the core components of BHD and genes were subjected to bulk molecular docking. The 3D structures (SDF format) of potentially active compounds were obtained from the PubChem database (https://pubchem.ncbi.nlm.nih.gov/) [30]. And save it as PDBQT format through AutoDock Tools 1.5.6. The 3D structures (PDB format) of the targets were obtained from the RCSB-PDB database (http://www.RCSB.org/) [31]. Use AutoDock Tools to remove water molecules, separate proteins, add nonpolar hydrogen, calculate the Gasteiger charge of the structure, and save it as a PDBQT file. The targets were used as receptors and set to be rigid. The compounds were used as ligands and set to be flexible. AutoDock Vina 1.1.2 was used to perform bulk molecular. The active sites for docking were determined by adjusting the original ligands on the different receptors by setting up a grid box size of 15 × 15 × 15 points with a spacing of 1.0 Å between grid points, covering almost the entire range of favourable protein binding sites. Each set of ligands and receptors produced 9 conformations. Collect the binding energy of the best-affinity conformation for a heatmap. The pocket structures and specific docking conformations of top 3 ligands bound to top 3 targets were visualized by PyMol 2.5 [32].

## 3. Results

### 3.1. Major Active Component Screen and Target Prediction for BHD

**Figure 1:**
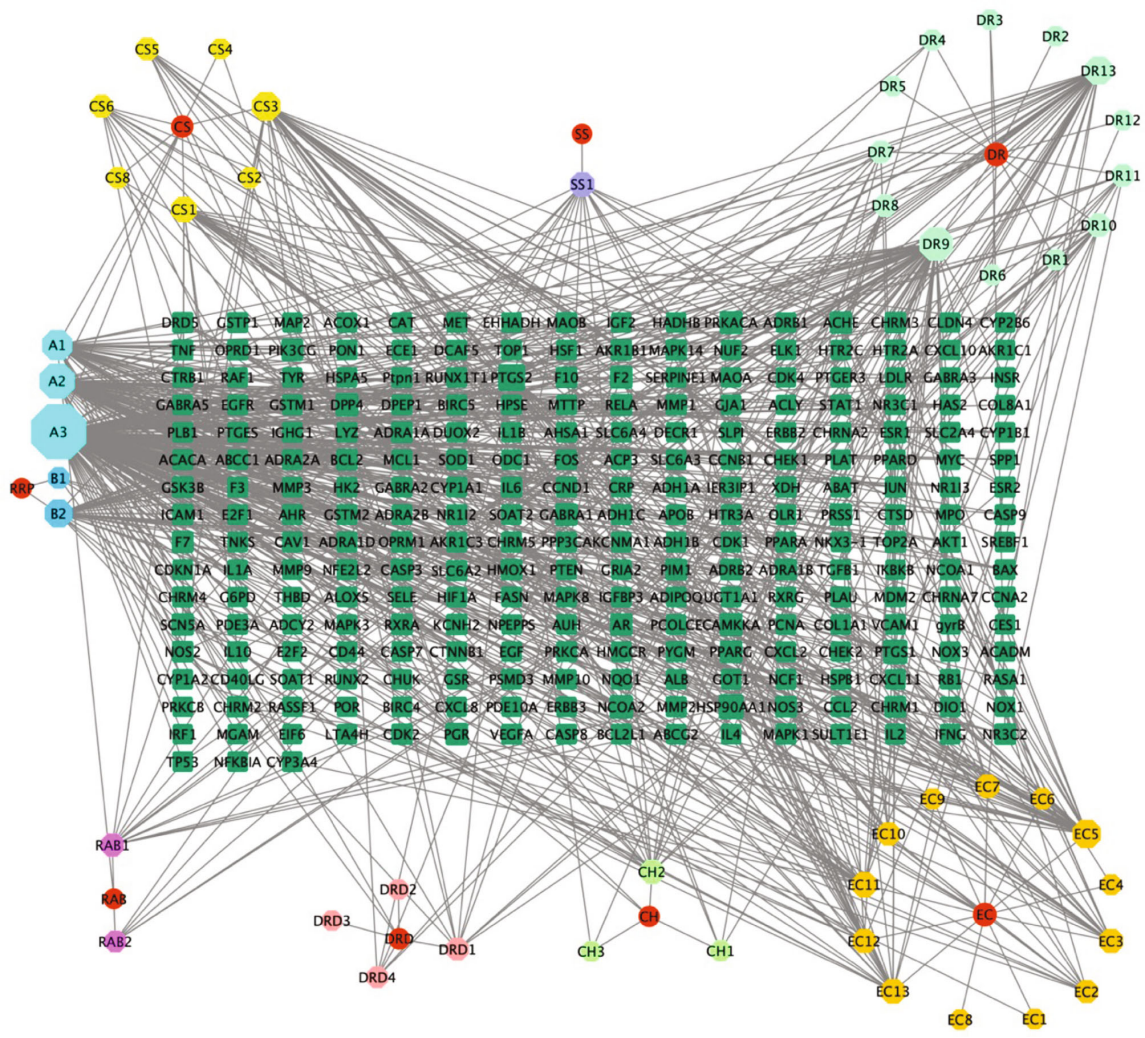
“Herbs-Components-Targets” network of BHD.

As shown in Table 1, sixty-two active ingredients of BHD were found in TCMSP database, including Cuscutae Semen (CS, 11 compounds), Rehmanniae Radix Praeparata (RRP, 2 compounds), Eucommiae Cortex (EC, 16 compounds), Dipsaci Radix (DRD, 6 compounds), Cistanches Herba (CH, 5 compounds), Drynariae Rhizoma (DR, 16 compounds), Sinapis Semen (SS, 3 compounds), and Radix Angelicae Biseratae (RAB, 3 compounds). A total of 51 active ingredients were obtained after weight removal (Table 1). A total of 752 targets were obtained for the active compounds of BHD: CS (344 targets), RRP (34 targets), EC (156 targets), DRD (18 targets), CH (22 targets), DR (141 targets), SS (20 targets), and RAB (17 targets). After removing duplications, 259 targets were obtained (Figure 1). Quercetin, luteolin, kaempferol, and aureusidin were identified as the key components of BHD.

### 3.2. BHD-BM PPI Interaction Network Analysis

**Figure 2:**
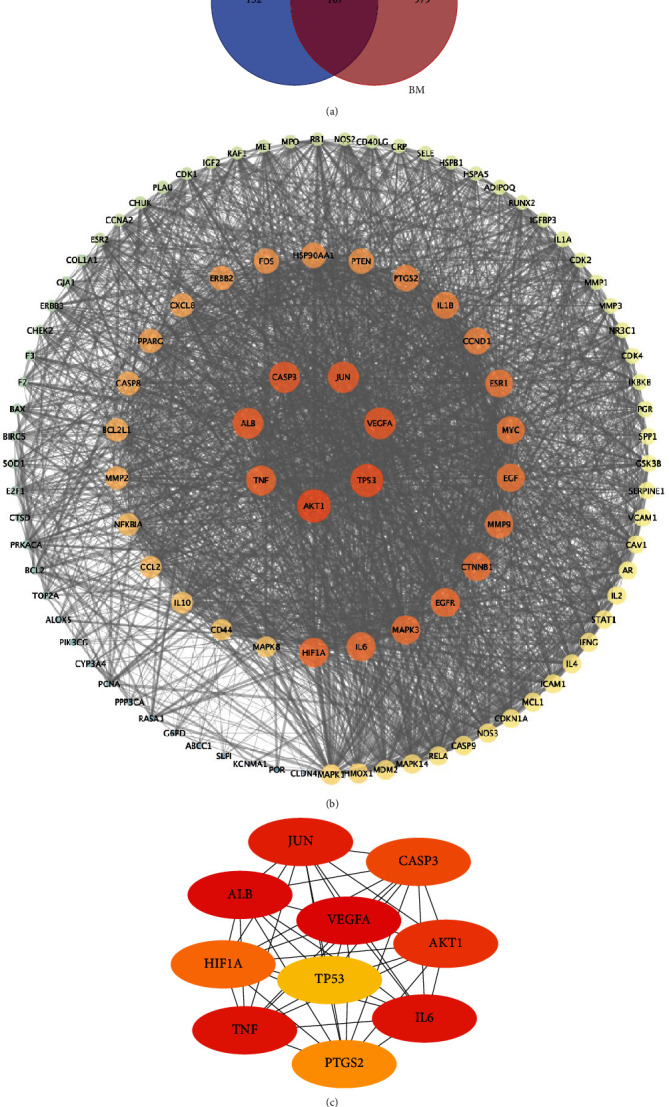
PPI network of BHD-BM. (a) Venn diagram shows the 107 genes associated with both BHD and BM. (b) PPI network built based on 107 BHD-BM genes. The center of the circle and the larger nodes represent more important hub nodes. (c) Topological analysis identified the hub genes of BHD-BM genes.

Database searching found 8,691 nonduplicated BM-related genes. By setting the degree score greater than the median three times, 1086 targets were finally obtained. Combined with the target screened for BHD active compounds in TCMSP dataset, 107 genes associated with both BHD and BM were considered to build the BHD-BM PPI interaction network (Figure 2(a)). PPI network of BHD-BM genes was constructed by STRING 11 and Cytoscape (Figure 2(b)). To narrow down the candidate target genes, the network was further analyzed by the Cytohubba plugin in Cytoscape. VEGFA, ALB, IL6, TNF, JUN, AKT1, CASP3, HIF1A, PTGS2, and TP53 were identified as the hub genes in the BHD-BM network (Figure 2(c)).

### 3.3. GO and KEGG Enrichment Analyses

**Figure 3:**
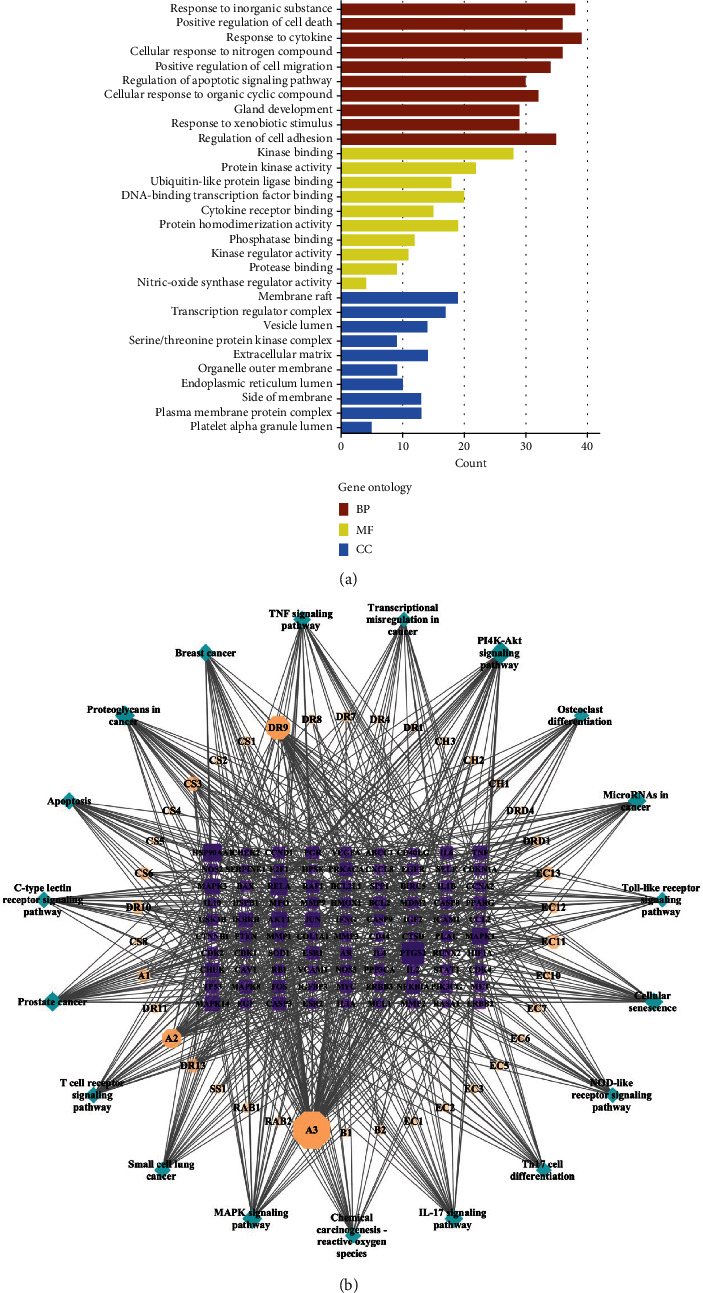
GO and KEGG enrichment analyses of BHD-BM. (a) GO enrichment analysis of BHD-BM genes in biological process (BP), cellular components (CC), and molecular function (MF). (b) BHD compounds, BM targets, and pathway network constructed by Cytoscape using the top 20 signaling pathways. The square nodes in the center represent the BHD-BM genes, the octagonal nodes in the middle ring represent the active components of BHD, and the outermost diamonds represent the related pathways.

GO enrichment analysis found 1,970 terms. Multiple targets of BHD were closely related to BM. The major biological process enriched for BHD-BM has a response to an inorganic substance, positive regulation of cell death, response to cytokine, positive regulation of cell migration, and regulation of apoptotic signaling pathway. The molecular function related to BHD-BM has kinase binding, protein kinase binding, ubiquitin-like protein ligase binding, DNA-binding transcription factor binding, and cytokine receptor binding. BHD-BM-related cellular components have membrane raft, transcription regulator complex, vesicle lumen, serine/threonine protein kinase complex, and extracellular matrix (Figure 3(a)). KEGG pathway enrichment found 164 terms. The main pathway relative to HBD-BM included PI3K-Akt signaling pathway, proteoglycans in cancer, MAPK signaling pathway, IL-17 signaling pathway, and microRNAs in cancer (Table 2).  Figure 3(b) shows the BHD compound, BM target, and pathway network constructed by Cytoscape using the top 20 signaling pathways.

### 3.4. Component-Target Docking Analysis

**Figure 4:**
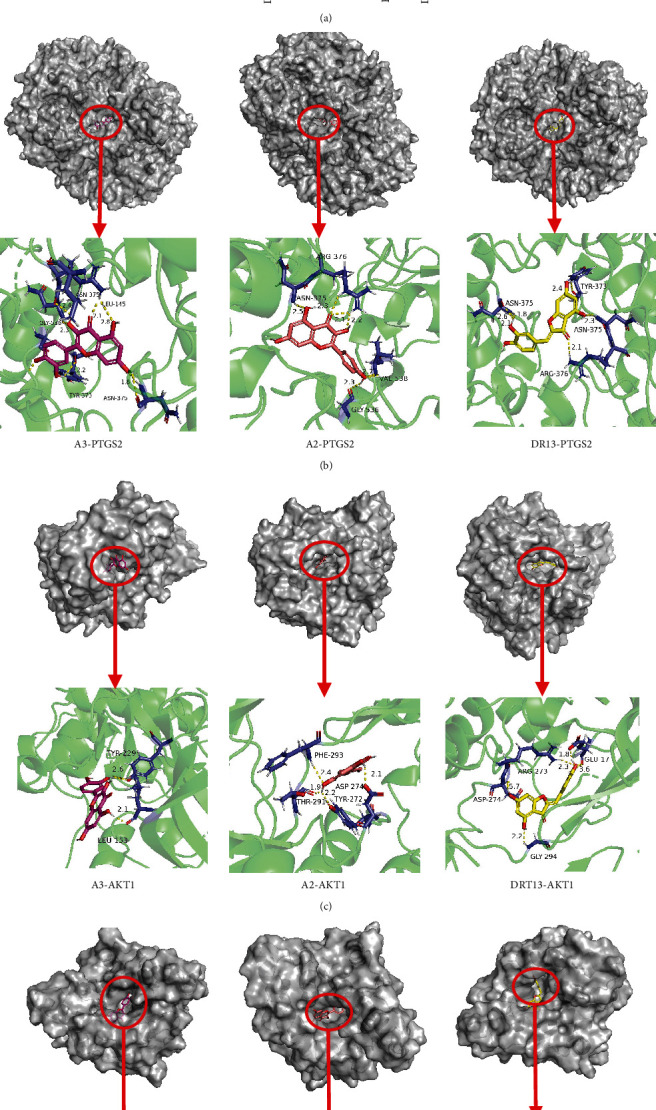
(a) Binding energy of the core compounds of BHD combined with the core genes. The docking complex of targets and their strongest binding components: (b) PTGS2, (c) AKT1, and (d) HSP9011A. The colored sticks represent the ligand, the spheres represent the protein structure, and the active site residues are shown.

According to the NTP analysis, the core compounds (Table 3) A3 (quercetin), A2 (kaempferol), DR13 (aureusidin), DR9 (luteolin), A1(beta-sitosterol), EC13 (syringetin), DR10 (xanthogalenol), and B2 (stigmasterol) were chosen for molecular docking verification with the core genes (Table 4) PTGS2, HSP90AA1, RELA, MAPK1, AKT1, MAPK3, JUN, MAPK14, CHUK, PRKACA, TNF, NFKBIA, TP53, IL6, CASP3, and VEGFA. The molecular docking results showed that the binding energies of the core components to the core genes were both negative, indicating that they can bind autonomously and tightly (Figure 4(a). Visualize the molecular docking results of the top 3 core compounds with the top 3 core genes by PyMol (Figures 4(b)–4(d)). Of these, quercetin was the most closely related to PTGS2, with five hydrogen bonds formed with the PTGS2 amino acid residues GLY-536, ASN-375, LEU-145, TYR-373, and ASN-375.

## 4. Discussion

Bone is one of the common sites of cancer metastasis. The unique structure and the dynamic metabolism pattern in bone make bone metastasis a different mode of malignant tumor metastasis. The mechanism of BM is still not very clear yet.

The “seed and soil” theory for tumor metastasis proposed by Paget in 1889 believed that the spread of tumor cells is determined by the interaction between cancer cells (seed) and host organs (soil) [33]. After more than a century's research, it is well recognized that the occurrence, development, and metastasis of tumors are not linear but involve multiple parallel overlapping routes [34, 35]. Bone microenvironment (BME) and tumor microenvironment (TME) are important factors for bone metastasis [36]. BME is composed by bone osteoblast cell, osteoclast cell, osteogenic cell, osteocyte cell, chondrocytes, adipocytes, fibroblasts, hematopoietic and mesenchymal stem cells, bone matrix, and various growth factors IGFs, BMPs, TGF*β*1, PDGFs, etc. [37]. TME includes immune cells, blood vessels, extracellular matrix (ECM), fibroblasts, lymphocytes, bone marrow-derived inflammatory cells, and signaling molecules. Interactions between tumor cells and nontumor cells in TME play important roles in cancer development, progression, and metastasis [38]. Bone marrow is an important hematopoietic organ and immune organ. In addition, immune cells have multiple interactions with bone marrow stromal cells [39]. The crosstalk between BME and TME may promote the metastatic potential of tumor cells.

The interaction between TME and BME involves multiple pathways as a comprehensive network. However, during the drug development process, it is very common to identify or design pharmacologically effective agents specifically targeting a single molecule or site. Drugs that act on a single molecular target often show unsatisfactory therapeutic effects or have large side effects when used to treat complex diseases in clinical. Therefore, drug development has gradually shifted from a “single target and single drug” model to a “network target multicomponent therapy” model [40, 41]. Multicomponents contained in Chinese medicine prescriptions (CMP) together with their targets constitute a huge drug interaction network. Due to this reason, CMP maybe not as good as the chemical drugs in the efficiency, which brings challenges to the evidence-based medicine verification of CMP. Network pharmacology is a bioinformatic network construction and network topology analysis strategy based on high-throughput data analysis, virtual computing, and network database retrieval [42]. It provides the possibility to study the complex network of CMP components-targets.

In this study, a total of 51 active components and 259 targets for BHD and 1086 BM targets were obtained. Combination of these two dataset, 107 BHD-BM intersection targets, was obtained finally. PPI interaction network analysis obtained the hub targets. GO and KEGG enrichment analyses showed that multiple targets of active components of BHD were closely related to BM. Membrane raft, transcription regulator complex, vesicle lumen, serine/threonine protein kinase complex, and extracellular matrix are the top cellular components found in GO and KEGG enrichment analyses. These cellular components are closely related to the formation of extracellular vesicles (EVs). EVs include exosomes, activation- or apoptosis-induced macrovesicles, and apoptotic bodies, which are key players in intercellular communication [43]. EVs are a heterogeneous group of membrane-limited vesicles loaded with lipids, various proteins, and multiple genetic materials such as DNAs, mRNAs, and noncoding RNAs [44]. Myeloid-derived suppressor cells (MDSC) are a heterogeneous population of immature myeloid cells with immunosuppressive activity. Tumor cells and stromal cells regulate the activation and expansion of MDSCs in the TME through EVs, thereby affecting the body's antitumor immune response [45].

KEGG enrichment analysis of BHD-BM showed that BHD compounds can affect BM through various pathways including regulation of apoptosis, immunity, inflammatory response, and treatment of primary tumors. A3 (quercetin), A2 (kaempferol), DR13 (aureusidin), DR9 (luteolin), A1 (beta-sitosterol), EC13 (syringetin), DR10 (xanthogalenol), and B2 (stigmasterol), the top 8 active components of BHD, were obtained after the bioinformatic analysis. Their combination to core targets was verified by molecular docking, which confirmed the multitarget and multichannel therapeutic effect of BHD on BM. PI3K-Akt signaling pathway was identified as one of the top signaling pathways in BHD-BM network. PI3K-Akt could participate in multiple cellular events to promote tumor initiation, progression, and metastasis. PI3K/Akt significantly increases bone metastases and osteolytic bone lesions in prostate cancer by mediating the stabilization of histone methyltransferase WHSC1 [46]. TLRs are key pathogen-sensing receptors to promote development and activation of the immune system. In TME, TLRs are widely expressed in the tumor-infiltrating immune cells and have critical roles in the prometastasis inflammatory microenvironment [47]. The activated TLR2 : TLR6 can induce TNF-alpha secretion by myeloid cells and enhance lung cancer bone metastasis growth [48]. Notably, IL-17 signaling pathway, T cell receptor signaling pathway, Th17 cell differentiation, and osteoclast differentiation enriched for BHD-BM were closely related to bone marrow-mediated immune activity. Homeostasis of osteoblasts and osteoclasts maintains bone stability. IL-17 is a marker for Th17 cells, a special subset of CD4^+^ helper T cells [49]. Th17 cells are positively associated with osteoclast formation in patients with myeloma [50]. By blocking the differentiation and function of Th17 cells, bone marrow mesenchymal stem cells can improve multiple sclerosis in an experimental autoimmune encephalomyelitis model [51]. All this evidence suggested that BHD may regulate bone marrow-mediated immunity by affecting Th17 cells.

In this study, we built the network between BHD compounds and BM target and screened and verified the in vivo combination of BHD components and their target employing network pharmacology and molecular docking analysis. Network pharmacology showed that quercetin and kaempferol were the top 2 core compounds of BHD.

Quercetin and kaempferol, as natural bioflavonoids, are widely found in a large number of natural herbs and have broad-spectrum antitumor effects [52, 53].

Animal and cellular experiments have shown that quercetin and kaempferol could downregulate AKT1, PTGS2, and VEGFA expressions [54–77] and upregulate TP53 expression [62, 71, 78–85] in various malignancies including breast cancer, prostate cancer, and non-small-cell lung cancer, which were the top 3 malignancies most likely to develop bone metastases [4–6]. This initially confirms the potential value of BHD in treating BM through their primary cause.

## 5. Conclusion

This study established the foundation of the new strategy for the pharmacology study of Chinese medicine. This study shows that the main active components of BHD can participate in the regulation of tumor and bone microenvironment through extracellular vesicles and affect the occurrence and development of malignant tumor bone metastases through various mechanisms such as immunity, apoptosis, and inflammation. It provides a basis for the study of the molecular mechanism of BHD and provides a new option for the treatment of bone metastases.

## Figures and Tables

**Table 1:** Major active components of BHD from TCMSP dataset.

Herb	Mark	Component
Cuscutae Semen	CS1	Sesamin
	CS2	NSC63551
	CS3	Isorhamnetin
	CS4	Campest-5-en-3beta-ol
	CS5	Isofucosterol
	CS6	Matrine
	CS7	Sophranol
	CS8	CLR
	A1	Beta-sitosterol
	A2	Kaempferol
	A3	Quercetin

Rehmanniae Radix Praeparata	B1	Sitosterol
	B2	Stigmasterol
Eucommiae Cortex	EC1	Mairin
	EC2	Erythraline
	EC3	3-Beta-hydroxymethyllenetanshiquinone
	EC4	Eucommin A
	EC5	(-)-Tabernaemontana
	EC6	Cyclopamine
	EC7	Dehydrodiconiferyl alcohol 4, gamma′-di-O-beta-D-glucopyranoside_qt
	EC8	GBGB
	EC9	Helenalin
	EC10	4-[(2S,3R)-5-[(E)-3-Hydroxyprop-1-enyl]-7-methoxy-3-methylol-2,3-dihydrobenzofuran-2-yl]-2-methoxy-phenol
	EC11	Beta-carotene
	EC12	(E)-3-[4-[(1R,2R)-2-Hydroxy-2-(4-hydroxy-3-methoxy-phenyl)-1-methylol-ethoxy]-3-methoxy-phenyl]acrolein
	EC13	Syringetin
	A1	Beta-sitosterol
	A2	Kaempferol
	A3	Quercetin

Dipsaci Radix	DRD1	Gentisin
	DRD2	Japonine
	DRD3	Sylvestroside III
	DRD4	Sylvestroside III_qt
	A1	Beta-sitosterol
	B1	Sitosterol

Cistanches Herba	CH1	Arachidonate
	CH2	Suchilactone
	CH3	Marckine
	A1	Beta-sitosterol
	A3	Quercetin

Drynariae Rhizoma	DR1	22-Stigmasten-3-one
	DR2	Marioside_qt
	DR3	Cycloartenone
	DR4	Digallate
	DR5	Cyclolaudenol
	DR6	Cyclolaudenol acetate
	DR7	Eriodictyol
	DR8	Eriodyctiol (flavanone)
	DR9	Luteolin
	DR10	Xanthogalenol
	DR11	(2R)-5,7-Dihydroxy-2-(4-hydroxyphenyl)chroman-4-one
	DR12	Naringenin
	DR13	Aureusidin
	A1	Beta-sitosterol
	B2	Stigmasterol

Sinapis Semen	SS1	Sinoacutine
	SS2	2-(2-Phenylethyl)-6-[[(5S,6R,7R,8S)-5,6,7-trihydroxy-4-keto-2-(2-phenylethyl)-5,6,7,8-tetrahydrochromen-8-yl]oxy]chromone
	SS3	Uniflex BYO

Radix Angelicae Biseratae	RAB1	Angelicone
	RAB2	Nodakenin
	A1	Beta-sitosterol

**Table 2:** KEGG enrichment analysis of BHD-BM module.

GO	Description	Count	Log *P*	Hits
hsa04151	PI3K-Akt signaling pathway	35	-38.739	AKT1, CCND1, BCL2, BCL2L1, CASP9, CDK2, CDK4, CDKN1A, CHUK, COL1A1, EGF, EGFR, ERBB2, ERBB3, GSK3B, HSP90AA1, IGF2, IKBKB, IL2, IL4, IL6, MCL1, MDM2, MET, MYC, NOS3, PIK3CG, MAPK1, MAPK3, PTEN, RAF1, RELA, SPP1, TP53, and VEGFA
hsa05205	Proteoglycans in cancer	29	-36.389	AKT1, CCND1, CASP3, CAV1, CD44, CDKN1A, COL1A1, MAPK14, CTNNB1, EGFR, ERBB2, ERBB3, ESR1, HIF1A, IGF2, MDM2, MET, MMP2, MMP9, MYC, PLAU, PRKACA, MAPK1, MAPK3, RAF1, TNF, TP53, VEGFA, and HPSE
hsa05215	Prostate cancer	28	-44.178	AKT1, AR, CCND1, BCL2, CASP9, CDK2, CDKN1A, CHUK, CTNNB1, E2F1, EGF, EGFR, ERBB2, GSK3B, HSP90AA1, IKBKB, MDM2, MMP3, MMP9, NFKBIA, PLAU, MAPK1, MAPK3, PTEN, RAF1, RB1, RELA, and TP53
hsa04010	MAPK signaling pathway	28	-30.404	AKT1, CASP3, CHUK, MAPK14, EGF, EGFR, ERBB2, ERBB3, FOS, HSPB1, IGF2, IKBKB, IL1A, IL1B, JUN, MET, MYC, PPP3CA, PRKACA, MAPK1, MAPK3, MAPK8, RAF1, RASA1, RELA, TNF, TP53, and VEGFA
hsa04657	IL-17 signaling pathway	25	-38.472	CASP3, CASP8, CHUK, MAPK14, FOS, GSK3B, HSP90AA1, IFNG, IKBKB, IL1B, IL4, IL6, CXCL8, JUN, MMP1, MMP3, MMP9, NFKBIA, MAPK1, MAPK3, MAPK8, PTGS2, RELA, CCL2, and TNF
hsa04218	Cellular senescence	25	-32.725	AKT1, CCND1, CCNA2, CDK1, CDK2, CDK4, CDKN1A, MAPK14, E2F1, IGFBP3, IL1A, IL6, CXCL8, MDM2, MYC, SERPINE1, PPP3CA, MAPK1, MAPK3, PTEN, RAF1, RB1, RELA, TP53, and CHEK2
hsa05206	MicroRNAs in cancer	25	-25.295	CCND1, BCL2, CASP3, CD44, CDKN1A, E2F1, EGFR, ERBB2, ERBB3, HMOX1, IKBKB, MCL1, MDM2, MET, MMP9, ABCC1, MYC, PLAU, MAPK1, MAPK3, PTEN, PTGS2, RAF1, TP53, and VEGFA
hsa04668	TNF signaling pathway	23	-32.674	AKT1, CASP3, CASP8, CHUK, MAPK14, FOS, ICAM1, IKBKB, IL1B, IL6, JUN, MMP3, MMP9, NFKBIA, MAPK1, MAPK3, MAPK8, PTGS2, RELA, CCL2, SELE, TNF, and VCAM1
hsa05224	Breast cancer	23	-29.847	AKT1, BAX, CCND1, CDK4, CDKN1A, CTNNB1, E2F1, EGF, EGFR, ERBB2, ESR1, ESR2, FOS, GSK3B, JUN, MYC, PGR, MAPK1, MAPK3, PTEN, RAF1, RB1, and TP53
hsa04210	Apoptosis	22	-28.889	AKT1, BIRC5, BAX, BCL2, BCL2L1, CASP3, CASP8, CASP9, CHUK, CTSD, FOS, IKBKB, JUN, MCL1, NFKBIA, MAPK1, MAPK3, MAPK8, RAF1, RELA, TNF, and TP53
hsa04625	C-Type lectin receptor signaling pathway	21	-29.677	AKT1, CASP8, CHUK, MAPK14, IKBKB, IL1B, IL2, IL6, IL10, JUN, MDM2, NFKBIA, PPP3CA, MAPK1, MAPK3, MAPK8, PTGS2, RAF1, RELA, STAT1, and TNF
hsa04660	T cell receptor signaling pathway	21	-29.677	AKT1, CD40LG, CDK4, CHUK, MAPK14, FOS, GSK3B, IFNG, IKBKB, IL2, IL4, IL10, JUN, NFKBIA, PPP3CA, MAPK1, MAPK3, MAPK8, RAF1, RELA, and TNF
hsa05222	Small-cell lung cancer	21	-30.829	AKT1, BAX, CCND1, BCL2, BCL2L1, CASP3, CASP9, CDK2, CDK4, CDKN1A, CHUK, E2F1, IKBKB, MYC, NFKBIA, NOS2, PTEN, PTGS2, RB1, RELA, and TP53
hsa05208	Chemical carcinogenesis—reactive oxygen species	20	-21.114	AKT1, CHUK, MAPK14, EGF, EGFR, FOS, HIF1A, HMOX1, IKBKB, JUN, MET, NFKBIA, MAPK1, MAPK3, MAPK8, PTEN, RAF1, RELA, SOD1, and VEGFA
hsa04659	Th17 cell differentiation	19	-25.608	CHUK, MAPK14, FOS, HIF1A, HSP90AA1, IFNG, IKBKB, IL1B, IL2, IL4, IL6, JUN, NFKBIA, PPP3CA, MAPK1, MAPK3, MAPK8, RELA, and STAT1
hsa04621	NOD-like receptor signaling pathway	19	-21.190	BCL2, BCL2L1, CASP8, CHUK, MAPK14, HSP90AA1, IKBKB, IL1B, IL6, CXCL8, JUN, NFKBIA, MAPK1, MAPK3, MAPK8, RELA, CCL2, STAT1, and TNF
hsa04620	Toll-like receptor signaling pathway	18	-24.128	AKT1, CASP8, CHUK, MAPK14, FOS, IKBKB, IL1B, IL6, CXCL8, JUN, NFKBIA, MAPK1, MAPK3, MAPK8, RELA, SPP1, STAT1, and TNF
hsa04380	Osteoclast differentiation	18	-22.496	AKT1, CHUK, MAPK14, FOS, IFNG, IKBKB, IL1A, IL1B, JUN, NFKBIA, PPARG, PPP3CA, MAPK1, MAPK3, MAPK8, RELA, STAT1, and TNF
hsa05202	Transcriptional misregulation in cancer	18	-19.335	BAX, BCL2L1, RUNX2, CCNA2, CDKN1A, IGFBP3, IL6, CXCL8, MDM2, MET, MMP3, MMP9, MPO, MYC, PLAU, PPARG, RELA, and TP53
hsa04115	p53 signaling pathway	17	-25.120	BAX, CCND1, BCL2, BCL2L1, CASP3, CASP8, CASP9, CDK1, CDK2, CDK4, CDKN1A, IGFBP3, MDM2, SERPINE1, PTEN, TP53, and CHEK2

**Table 3:** The network topology parameters of the top 8 components of BHD, based on BHD compounds, BM targets, and pathway network.

Mol ID	Mark	Component	Degree	Betweenness centrality	Closeness centrality
MOL000098	A3	Quercetin	71	0.27068666	0.57086614
MOL000422	A2	Kaempferol	27	0.06990185	0.44207317
MOL001978	DR13	Aureusidin	14	0.01494905	0.39459459
MOL000006	DR9	Luteolin	38	0.06739532	0.45886076
MOL000359	A1	Beta-sitosterol	11	0.01825399	0.40055249
MOL011604	EC13	Syringetin	11	0.00904134	0.38770053
MOL009091	DR10	Xanthogalenol	5	0.00086333	0.35194175
MOL000449	B2	Stigmasterol	4	0.00564496	0.36616162

**Table 4:** The network topology parameters of the top 16 targets, based on BHD compounds, BM targets, and pathway network.

Target	Degree	Betweenness centrality	Closeness centrality
PTGS2	32	0.14759775	0.51056338
HSP90AA1	24	0.06347734	0.48013245
RELA	22	0.02782304	0.47697368
MAPK1	20	0.01764473	0.47077922
AKT1	19	0.01811404	0.47077922
MAPK3	18	0.01261661	0.45886076
JUN	17	0.01429791	0.45031056
MAPK14	16	0.01521324	0.41666667
CHUK	15	0.00677473	0.42397661
PRKACA	14	0.02579619	0.44753086
TNF	14	0.00973159	0.44478528
NFKBIA	14	0.0060302	0.42151163
TP53	13	0.00903582	0.453125
IL6	12	0.00691109	0.41907514
CASP3	10	0.01476703	0.453125
VEGFA	8	0.00287182	0.41428571

## Data Availability

All the data can be obtained from the open-source website we provide, and the conclusion can be drawn through the analysis of the relevant software.
